# 3D Poisson-Based Neighborhood Capacity Analysis for Millimeter Wave Communications

**DOI:** 10.3390/s22062098

**Published:** 2022-03-08

**Authors:** Massimiliano Comisso, Giulia Buttazzoni, Stefano Pastore, Francesca Vatta, Fulvio Babich

**Affiliations:** Department of Engineering and Architecture, University of Trieste, Via A. Valerio 10, 34127 Trieste, Italy; gbuttazzoni@units.it (G.B.); pastore@units.it (S.P.); vatta@units.it (F.V.); babich@units.it (F.B.)

**Keywords:** millimeter waves, Poisson point process, neighbor order, 3D analysis, link capacity

## Abstract

This paper proposes a theoretical model for evaluating the capacity of a millimeter wave (mmWave) source destination link when the nodes are distributed according to a three-dimensional (3D) homogeneous Poisson point process. In the presented analysis, different from the existing approaches, the destination lies in an arbitrary location with respect to the source; thus, the link performance can be evaluated for a neighbor of any order. Moreover, the developed model relies on a realistic propagation environment, characterized by path loss attenuation and shadowing in line of sight (LoS), non-LoS, and outage link state conditions. The derived formulas, which are calculated in closed-form and validated by independent Monte Carlo simulations, are used to investigate the influence of the intensity parameter, of the antenna gain, and of the mmWave frequency band on the link capacity for any possible neighbor in a practical 3D scenario.

## 1. Introduction

The transition towards the fifth-generation (5G) cellular system at present mainly concerns the implementation of the evolved network function virtualization architecture and, in many cases, the preliminary deployment of novel transceivers operating in the 3.7 GHz band [1]. However, the final task will be accomplished in upcoming years when the radio components are targeted to extremely high frequencies with the aim of exploiting the presently underutilized millimeter wave (mmWave) bands [2,3,4,5]. This latter element, combined with the densification of the base stations (BSs) [6,7,8,9], the integration between the 5G and the IEEE 802.11ad gigabit wireless fidelity systems [10,11,12,13], and the usage on each device of massive, electrically-small antenna arrays [14,15,16,17], represent the actual technological jump toward a pervasive terrestrial network, capable of supporting a huge range of applications, including massive data acquisition and low-latency multimedia streaming [18,19,20].

For this reason, several 5G proposals and mathematical frameworks have been recently considered to address the problems related to the feasibility of such an evolved system. Among these problems, great importance is assumed by the antenna steering between BS and users during the initial access [21], the compensation of the relative beam pointing errors [22], the possibility of adopting a pseudowire link model instead of an interference-limited one [23], the data link layer algorithms to guarantee massive multiple access [24], and the derivation of realistic coverage probability estimations [25]. The usual assumption adopted for treating all of these problems from a theoretical perspective consists in considering the nodes distributed according to a homogeneous Poisson point process (PPP), since this spatial statistic is more suitable to obtain manageable mathematical models while maintaining a satisfactory level of applicability to real network deployments [26]. Within this approach, and still for tractability purposes, the conceived theories usually rely on the nearest neighbor assumption to probabilistically model the distance between a pair of nodes. Unfortunately, this hypothesis might become unrealistic in the mobile ultra-dense scenarios expected for the 5G application domain, where any node, regardless of its greater or lesser proximity to another station, may be a target for that station as a source, destination, or interferer. This aspect may be taken into account in combination with a second positive element, consisting in the availability of spatial statistics, including neighbors of any order, which have been derived both for the two-dimensional (2D) [27] and three-dimensional (3D) environments [28]. A deeper characterization of the coverage properties of the mmWave links for farther neighbors has been partly addressed in [29], but limiting the investigation to the 2D domain in line of sight (LoS) conditions. A more complete view involving the general 3D case would be instead of considerable interest, not only for completeness of the treatise, but mainly for the realism of the scenario. The expected small size of the 5G cells, with BSs even placed between the floors of a building, can in fact lead to situations in which two nodes may reciprocally lie overground or underground and not exclusively side-by-side on an horizontal plane. By consequence, the development of a 3D mmWave coverage analysis, including the neighbors of any order, may represent a desirable advance for deepening the insights in the forthcoming 5G/6G networks.

To address this issue, this paper presents a 3D mathematical model for estimating the capacity of a mmWave communication when the nodes around a source are located in agreement with a PPP and the destination can be a neighbor of any order. The analysis, which is checked through independent Monte Carlo simulations, was developed, considering a realistic three-state link model accounting for OUTage (OUT), LoS, and non-LoS (NLoS) conditions in the presence of path loss attenuation and mid-scale fading. The obtained expressions, which are derived in closed-form, are used to explore the impact on the link capacity of the cell radius and of the transmitting/receiving antenna gain in the 28 and 73 GHz mmWave channels, with specific attention to the sensitivity of the performance, with respect to the neighbor order.

The paper is organized as follows. Section 2 introduces the addressed scenario. Section 3 presents the theoretical analysis. Section 4 discusses the numerical results. Finally, Section 5 summarizes the most relevant conclusions.

*Notation.* Throughout the paper, the following notation is used: $N_{>0}$ denotes the set of positive integers; $R_{>0}$ and $R_{\geq 0}$ denote the sets of positive and non-negative reals, respectively; $\delta_{i,j}$ denotes the Kronecker delta (i.e., $\delta_{i,j}=1$ if i=j, $\delta_{i,j}=0$ otherwise); $x^{+}$ denotes the positive part; ⌈x⌉ denotes the ceiling function; ${𝟙}_{X}\left(x\right)$ denotes the indicator function (i.e., ${𝟙}_{X}\left(x\right)\;=\;1$ if x∈X, ${𝟙}_{X}\left(x\right)\;=\;0$ otherwise); δ(x) denotes the Dirac delta function; erf(x) denotes the error function; Γ(x) denotes the Euler gamma function; Γ(·;x) and γ(·;x) denote the upper and the lower incomplete Euler gamma functions, respectively; F1F2(·;·,·;x) denotes the generalized hypergeometric function with one type-1 parameter and two type-2 parameters.

## 2. Scenario

Consider a 5G mmWave communication between a source S and a destination that is realized in an $R^{3}$ space having origin *O* coincident with the position of S. The destination $D_{k}$ for $k\in N_{>0}$ is identified by the *k*-th neighbor of S, whose location is described by a 3D homogeneous PPP of intensity λ. Thus, $D_{k}$ represents the nearest neighbor when k=1, the second nearest neighbor when k=2, the third nearest neighbor when k=3, etc. This statistical displacement allows one to model the $S-D_{k}$ distance by a random variable (RV) $R_{k}$ having probability density function (PDF) [28]:

(1) $$f_{R_{k}}\left(r\right)=\frac{{\left(4\pi \lambda \right)}^{k}}{3^{k-1}\Gamma \left(k\right)}\;r^{3k-1}\exp\left(-\frac{4}{3}\pi \lambda r^{3}\right){𝟙}_{R_{\geq 0}}\left(r\right),\;k\in N_{>0},$$

from which the corresponding cumulative distribution function (CDF) may be directly evaluated as:

(2) $$F_{R_{k}}\left(r\right)=\int_{-\infty }^{r}f_{R_{k}}\left(r^{\prime }\right)dr^{\prime }=\frac{1}{\Gamma (k)}\;\gamma \left(k;\frac{4}{3}\pi \lambda r^{3}\right){𝟙}_{\;R_{\geq 0}}\left(r\right),\;k\in N_{>0}.$$

As compared to the typically considered nearest neighbor case [6], the statistic in (2) allows one to model many realistic 3D communication scenarios in which the desired destination can lie in an arbitrary position, not necessarily coincident with the node closest to S. Reasonably, the occurrence of such events might not be unusual in forthcoming 5G networks, considering their expected ultra-density characteristics, which would considerably reduce the probability that a source destination pair exclusively involves the closest nodes.

In agreement with the link state model proposed in [2], the mmWave channel can be in OUT, LoS, or NLoS conditions. The first one describes the situation in which the communication is impossible because of a too high path loss. An event that may occur in large 5G cells. The LoS condition instead describes the scenario of optical visibility, while the NLoS one—that of non-optical visibility. When the link lies in one of these two latter states, the communication is possible. To statistically model the three situations, consider the RV *H*, which is conditionally dependent on the distance $R_{k}$ [8], and whose realizations, i.e., h=0 (OUT), h=1 (LoS), and h=2 (NLoS), include the three possible states. Accordingly, one can define the conditional probability mass function (PMF) of *H* given $R_{k}$ as:

(3) $$f_{H|R_{k}}\left(h|r\right)=\left\{\begin{array}{cc} \max[0,1-\exp\left(-a_{\mathrm{out}}r+b_{\mathrm{out}}\right)] & h=0\\ \left[1-f_{H|R_{k}}\left(0|r\right)\right]\exp\left(-a_{\mathrm{los}}r\right) & h=1\\ 1-f_{H|R_{k}}\left(0|r\right)-f_{H|R_{k}}\left(1|r\right) & h=2 \end{array}\right.,$$

where $a_{\mathrm{out}}$, $b_{\mathrm{out}}$, and $a_{\mathrm{los}}$ are constants dependent on the adopted mmWave band, which have been evaluated in [2] by combining experimental data and curve fitting procedures. An interesting aspect that can be immediately observed from (3) is the direct impact of the neighbor order. This latter quantity, in fact, does not only influence the $S-D_{k}$ distance by (2), but also the link state, since the probability of being in LoS conditions decreases with the statistical increase of *r* consequent to the increase of *k*.

For each link state, the propagation environment is characterized by the distance-dependent path loss attenuation and by the random power fluctuations due to mid-scale fading. Since it has been proved that, in the mmWave channel, small-scale power fluctuations have minor effects as compared to shadowing when local phenomena are not of interest [5] and fast fading is neglected. Path loss attenuation is described through the RV $L_{h}$, which is a function of the distance, and is hence expressed as [6]:

(4) $$L_{h}\left(R_{k}\right)=\left\{\begin{array}{cc} +\infty & h=0\\ \alpha_{h}R_{k}^{\beta_{h}} & h=1,2 \end{array}\right.,$$

where $\alpha_{h}$ denotes the floating-intercept and $\beta_{h}$ represents the average path loss exponent for h=1,2. Similar to the link state constants, the $\alpha_{h}$ and $\beta_{h}$ parameters have been characterized in [2] by performing extensive measurement campaigns in the 28 and 73 GHz mmWave bands, and then applying best-fit linear regression strategies for their estimation. Mid-scale fading is instead described, for h=1,2, by a log-normally distributed RV $\Xi_{h}$, having PDF [4]:

(5) $$f_{\Xi_{h}}\left(\xi \right)=\frac{1}{\sqrt{2\pi }\sigma_{h}\xi }\exp\left(-\frac{\log^{2}\xi }{2\sigma_{h}^{2}}\right){𝟙}_{R_{>0}}\left(\xi \right),$$

where $\sigma_{h}$ is the shadowing standard deviation for the respective link state. Concerning (5), it is worth to observe that mid-scale fading can be present even in LoS conditions (h=1), since the optical visibility does not directly imply the clear radio visibility. This second (more restrictive) condition requires the complete clearance of the first Fresnel zone of the link. When this condition is not met, and hence a partial radio obstruction of the LoS link is present, shadowing must be taken into account.

The noise power is the further element considered in the propagation model, here used to describe the mmWave channel. This quantity, which can be assumed constant, may be expressed as [6]:

(6) $$N=N_{0}\cdot W\cdot F,$$

where $N_{0}≅3.98\times 10^{-21}$ W/Hz identifies the noise spectral density, *W* denotes the mmWave receiver bandwidth and F its noise figure. As discussed in [23], the noise power has the crucial impact on the result of a mmWave communication as long as the commonly adopted “pseudowired” assumption holds. This assumption implies that the interference incoming from nodes different from the $S-D_{k}$ pair that operate in the same mmWave frequency band can be neglected when highly directional links are established. The availability of such links is allowed by the low carrier wavelengths that characterize the mmWave band, which allow the installation on 5G nodes of electrically-small arrays with tens or even hundreds of antennas. Under these conditions, the transmitting/receiving antenna patterns may be described by a flat-top model having as parameters a main lobe beam width Ω, a maximum gain *G*, and a backlobe gain *g*. To simplify the notation, these parameters are assumed independent of *k*, i.e., identical for all neighbor orders. To account for both the ideal case of perfect beam alignment and the more realistic one of imperfect alignment, the transmitting and receiving gains are respectively described by the RVs $G_{S}$ and $G_{D}$ having PDFs:

(7a) $$\begin{array}{cc} f_{G_{S}}\left(g_{S}\right) & =\Delta \;\delta \left(g_{S}-G\right)+\left(1-\Delta \right)\delta \left(g_{S}-g\right), \end{array}$$

(7b) $$\begin{array}{cc} f_{G_{D}}\left(g_{D}\right) & =\Delta \;\delta \left(g_{D}-G\right)+\left(1-\Delta \right)\delta \left(g_{D}-g\right), \end{array}$$

where:

(8) $$\Delta =\left\{\begin{array}{cc} 1 & \mathrm{perfect}\;\mathrm{alignment}\\ \mathrm{erf}{\left(\frac{\Omega }{2\sqrt{2}\hat{\sigma }}\right)}^{{}^{{}^{{}^{{}^{}}}}} & \mathrm{imperfect}\;\mathrm{alignment} \end{array}\right.,$$

in which $\hat{\sigma }$ represents the standard deviation of the zero-mean Gaussian beam steering error [6]. In particular, the first case in (8) leads to constant gains, corresponding to PDFs associated with degenerate distributions, while the second case leads to actual random gains. This formulation is adopted to jointly model both situations, making the analysis more compact.

## 3. Analysis

By relying on the system model described in the previous section, we now present the analysis required to derive the capacity of the $S-D_{k}$ link. The analysis consists of three steps. The first one considers the impact of path loss attenuation and shadowing for a given link state. The second step accounts for the influence of all the three possible link states. The third step evaluates the statistic of the signal to noise ratio (SNR), accounting for the antenna gains to finally obtain the link capacity.

As a first step, define, for the LoS and NLoS states, the RV:

(9) $$T_{h}=P_{S}L_{h}\left(R_{k}\right),\;h=1,2;\;k\in N_{>0},$$

where $P_{S}$ represents the transmission power. This RV describes the power received by $D_{k}$ from S in the absence of shadowing for a given link state when the communication is possible. Omnidirectional transmissions/receptions are at present assumed, since the impact of the antenna gains will be considered in the last step of the analysis. By using (4), one can express (9) in a more compact form as:

(10) $$T_{h}=\frac{P_{S}}{\alpha_{h}R_{k}^{\beta_{h}}}=\frac{\varpi_{h}}{R_{k}^{\beta_{h}}},\;h=1,2;\;k\in N_{>0},$$

where:

(11) $$\varpi_{h}=\frac{P_{S}}{\alpha_{h}}.$$

The CDF $F_{T_{h}}\left(t\right)$ of the RV $T_{h}$ can be derived by inverting (10) with respect to $R_{k}$, and then applying (2) to account for the neighbor location. These operations yield:

(12) $$\begin{array}{cc} F_{T_{h}}\left(t\right) & =\mathrm{Pr}\left\{T_{h}\leq t\right\}=\mathrm{Pr}\left\{\frac{\varpi_{h}}{R_{k}^{\beta_{h}}}\leq t\right\}=1-\mathrm{Pr}\left\{R_{k}<{\left(\frac{\varpi_{h}}{t}\right)}^{\frac{1}{\beta_{h}}}\right\}\\ & =1-F_{R_{k}}\left[{\left(\frac{\varpi_{h}}{t}\right)}^{\frac{1}{\beta_{h}}}\right]=\frac{\Gamma \left(k;\varphi_{h}t^{-\frac{3}{\beta_{h}}}\right)}{\Gamma (k)}{𝟙}_{R_{>0}}\left(t\right),\;h=1,2;\;k\in N_{>0}, \end{array}$$

where by (11):

(13) $$\varphi_{h}=\frac{4}{3}\pi \lambda \varpi_{h}^{\frac{3}{\beta_{h}}}=\frac{4}{3}\pi \lambda {\left(\frac{P_{S}}{\alpha_{h}}\right)}^{\frac{3}{\beta_{h}}}.$$

Now, to account for mid-scale fading, let us now consider the RV identified by the product:

(14) $$Q_{h}=T_{h}\;\Xi_{h},$$

whose CDF $F_{Q_{h}}\left(q\right)$ may be evaluated by using (5) and recalling the product distribution [30], which leads to:

(15) $$F_{Q_{h}}\left(q\right)=\int_{-\infty }^{+\infty }F_{T_{h}}\left(\frac{q}{\xi }\right)f_{\Xi }\left(\xi \right)d\xi =\frac{1}{\sqrt{2\pi }\sigma_{h}\Gamma \left(k\right)}\int_{0}^{+\infty }\Gamma \left[k;\varphi_{h}{\left(\frac{\xi }{q}\right)}^{\frac{3}{\beta_{h}}}\right]\frac{1}{\xi }\exp\left(-\frac{\log^{2}\xi }{2\sigma_{h}^{2}}\right)d\xi .$$

Unfortunately, this integral cannot be analytically solved, thus an alternative strategy is required. To this aim, one may usefully adopt the improved Gaussian approximation developed in [31], which can be applied to any product between two RVs when one of them follows a normal or a log-normal distribution. In particular, the usage of this approximation in (15) allows one to obtain [31]:

(16) $$F_{Q_{h}}\left(q\right)≅\frac{2}{3}\sum_{n=-1}^{1}\frac{1}{4^{\left|n\right|}}\;F_{T_{h}}\left(\frac{q}{\varepsilon_{h}^{n}}\right)=\left[\sum_{n=-1}^{1}A_{n,k}\Gamma \left(k;\chi_{n,h}q^{-\frac{3}{\beta_{h}}}\right)\right]{𝟙}_{R_{>0}}\left(q\right),\;h=1,2;\;k\in N_{>0},$$

where $\varepsilon_{h}=\exp\left(\sqrt{3}\sigma_{h}\right)$ and by (13):

(17a) $$A_{n,k}=\frac{2}{3\times 4^{\left|n\right|}\Gamma \left(k\right)},\;n=-1,0,1,$$

(17b) $$\chi_{n,h}=\varphi_{h}\varepsilon_{h}^{\frac{3n}{\beta_{h}}}=\frac{4}{3}\pi \lambda {\left(\frac{P_{S}\varepsilon_{h}^{n}}{\alpha_{h}}\right)}^{\frac{3}{\beta_{h}}},\;n=-1,0,1.$$

As a second step of the analysis, consider the joint impact of the three link states. To enable the analytical tractability of this step, lets firstly remove the conditioning of (3) with respect to $R_{k}$ in agreement with the neighbor distribution. This operation, which implies the independence between path loss attenuation and link state probability, represents the second approximation introduced in the developed model. To observe the impact of this approximation on the accuracy of the analysis, in the subsequent section the theoretical results will be compared to the numerical ones derived by Monte Carlo simulations. According to the adopted approach, the unconditional link state probability may be evaluated from:

(18) $$f_{H}\left(h\right)=\int_{-\infty }^{+\infty }f_{H|R_{k}}\left(h|r\right)f_{R_{k}}\left(r\right)dr.$$

By using (1) and (3) in (18) and performing some manipulations, one obtains:

(19) $$f_{H}\left(h\right)=\left\{\begin{array}{cc} \max{\left[0,1-\eta_{k}\left(a_{\mathrm{out}},b_{\mathrm{out}}\right)\right]}_{{}_{{}_{{}_{{}_{{}_{}}}}}} & h=0\\ \left[1-f_{H}\left(0\right)\right]\eta_{k}{\left(a_{\mathrm{los}},0\right)}_{{}_{{}_{{}_{{}_{{}_{}}}}}} & h=1\\ 1-f_{H}\left(0\right)-f_{H}\left(1\right) & h=2 \end{array}\right.,\;\;\;k\in N_{>0}$$

where:

(20) $$\eta_{k}\left(a,b\right)=\frac{e^{b}}{\Gamma (k)}\sum_{j=0}^{2}B_{j}\left(a\right)E_{j,k}\left(a\right),$$

with:

(21a) $$\begin{array}{cc} B_{j}\left(a\right) & =\frac{{\left(-a\right)}^{j}}{2^{\delta_{j,2}}}{\left(\frac{3}{4\pi \lambda }\right)}_{{}_{{}_{{}_{{}_{}}}}}^{\frac{j}{3}},\;j=0,1,2, \end{array}$$

(21b) Ej,k(a)=Γk+j3F1F2k+j3;2j3,2j+13−δj,2;−a336πλ, j=0,1,2.

This latter PMF enables to define the RV:

(22) $$P≅\left\{\begin{array}{cc} 0 & w.p.f_{H}\left(0\right)\\ Q_{1} & w.p.f_{H}{\left(1\right)}^{{}^{{}^{{}^{}}}}\\ Q_{2} & w.p.f_{H}{\left(2\right)}^{{}^{{}^{{}^{}}}} \end{array}\right.,$$

which identifies the power received by $D_{k}$ from S when all the three link states (OUT: h=0, LoS: h=1, NLoS: h=2) are taken into account. The CDF of *P* may be evaluated by using (16) and exploiting the concept of the mixture distribution [32], thus obtaining:

(23) $$F_{P}\left(p\right)≅\sum_{h=0}^{2}f_{H}\left(h\right)F_{Q_{h}}\left(p\right)=\left[f_{H}\left(0\right)+\sum_{h=1}^{2}\sum_{n=-1}^{1}f_{H}\left(h\right)A_{n,k}\Gamma \left(k;\chi_{n,h}p^{-\frac{3}{\beta_{h}}}\right)\right]{𝟙}_{R_{>0}}\left(p\right),\;k\in N_{>0},$$

where the degenerate distribution:

(24) $$F_{Q_{0}}\left(q\right)={𝟙}_{R_{>0}}\left(q\right),$$

is introduced to model the CDF of the RV $Q_{0}$, describing the null power received by $D_{k}$ when the link is in the OUT state.

The third and final step of the analysis begins by evaluating the CDF of the RV:

(25) $$Y=\frac{PG_{S}G_{D}}{N},$$

representing the SNR and in which the noise power N is given by (6), while the PDFs of the antenna gains are given by (7). Using these statistics, one can derive the PDF $f_{G}\left(g\right)$ of the RV:

(26) $$G=G_{S}G_{D},$$

representing the product gain. This task can be accomplished firstly adopting the product distribution referred to the PDF [30], and then exploiting the scaling and translation properties of the Dirac delta function, so as to obtain:

(27) $$f_{G}\left(g\right)=\int_{g}^{G}f_{G_{D}}\left(\frac{g}{g_{S}}\right)\frac{f_{G_{S}}\left(g_{S}\right)}{g_{S}}dg_{S}=\sum_{l=0}^{2}D_{l}\delta \left(g-G^{l}g^{2-l}\right),$$

where:

(28) $$D_{l}=\left(1+\delta_{l,1}\right)\Delta^{l}{\left(1-\Delta +\delta_{l,2}\right)}^{2-l},\;l=0,1,2.$$

As discussed at the end of the previous section, the noise-limited assumption [23] is adopted, thus the possible interference received by $D_{k}$ from transmitters different from S can be neglected thanks to the usage of high-directional antennas realized by arrays of several elements. The CDF $F_{Y}\left(\upsilon \right)$ of Y may be hence calculated by combining the scaling rule for a RV and again the product distribution referred to the CDF. By using (23) and (27), this operation leads to:

(29) $$\begin{array}{cc} F_{Y}\left(\upsilon \right) & =\int_{-\infty }^{+\infty }F_{P}\left(\frac{N\upsilon }{g}\right)f_{G}\left(g\right)dg=\sum_{l=0}^{2}D_{l}F_{P}\left(\frac{N\upsilon }{G^{l}g^{2-l}}\right)\\ & ≅\left[f_{H}\left(0\right)+\sum_{l=0}^{2}\sum_{h=1}^{2}\sum_{n=-1}^{1}f_{H}\left(h\right)A_{n,k}D_{l}\Gamma \left(k;\mu_{n,h,l}\upsilon^{-\frac{3}{\beta_{h}}}\right)\right]{𝟙}_{R_{>0}}\left(\upsilon \right),\;k\in N_{>0}, \end{array}$$

where by (17b):

(30) $$\mu_{n,h,l}=\chi_{n,h}\;{\left(\frac{N}{G^{l}g^{2-l}}\right)}^{-\frac{3}{\beta_{h}}}=\frac{4}{3}\pi \lambda {\left(\frac{P_{S}G^{l}g^{2-l}\varepsilon_{h}^{n}}{\alpha_{h}N}\right)}^{\frac{3}{\beta_{h}}},\;n=-1,0,1;\;h=1,2;\;l=0,1,2.$$

Remembering that the complementary CDF (CCDF) ${\bar{F}}_{Y}\left(\upsilon \right)=1-F_{Y}\left(\upsilon \right)=\mathrm{Pr}\left\{Y>\upsilon \right\}$ coincides with the coverage probability for the $S-D_{k}$ communication [33], the quantity in (29) can be directly exploited to finally evaluate the link capacity according to the occurring SNR. To provide a wider view regarding this aspect, two cases are addressed: a limiting one, obtained exploiting the Shannon bound, and another one, relying on a fixed Quadrature Phase-Shift Keying (QPSK) modulation. This latter scheme is modeled using the approximated formula considered in [34], which provides the following expression for the capacity of a mmWave link as a function of the SNR:

(31) $$C\left(\upsilon \right)=\left[1-F_{Y}\left(\upsilon \right)\right]\left\{\begin{array}{cc} \log_{2}{\left(1+\upsilon \right)}_{{}_{{}_{{}_{{}_{{}_{{}_{}}}}}}} & \mathrm{Shannon}\;\mathrm{bound}\\ 2{\left[1-\exp\left(\varsigma_{1}-\varsigma_{2}\upsilon^{\varsigma_{3}}\right)\right]}^{+} & \mathrm{QPSK}\;\mathrm{modulation} \end{array}\right.,$$

where $\varsigma_{1}≅0.0102$, $\varsigma_{2}≅0.6746$, $\varsigma_{3}≅0.9308$ [34]. It is interesting to observe that this latter result, which represents the objective of the analysis, as well as the intermediate ones, are all available in analytical form. Hence, their numerical estimation can be obtained using the procedures commonly implemented in the mathematical tools developed for the approximation of the special functions, thus limiting the computational cost with respect to the numerical integrations required when closed-forms are not available.

## 4. Results and Discussion

The results derived from the proposed framework are calculated by using the parameters shown in Table 1, which are referred to the experimental measurement campaign realized in [2] for the 28 and 73 GHz channels. For each considered scenario, the intensity λ of the homogeneous PPP is inferred from the average cell radius [6]:

(32) $$\rho =\frac{1}{\sqrt{\pi \lambda }}.$$

All theoretical formulas and validation tools are implemented in MATLAB, adopting a nonuniform discretization of the support of the modeled RVs in order to limit the processing time necessary to compute the corresponding statistics. The presented results are organized in five subsections. The first subsection is devoted to the validation of the developed model. The second and the third subsections are focused on the impact of the cell radius and of the maximum product gain. The fourth subsection investigates the influence of the beam alignment error, while the latter subsection compares the Shannon capacity with that obtained using the QPSK modulation.

### 4.1. Model Validation

**Figure 1:**
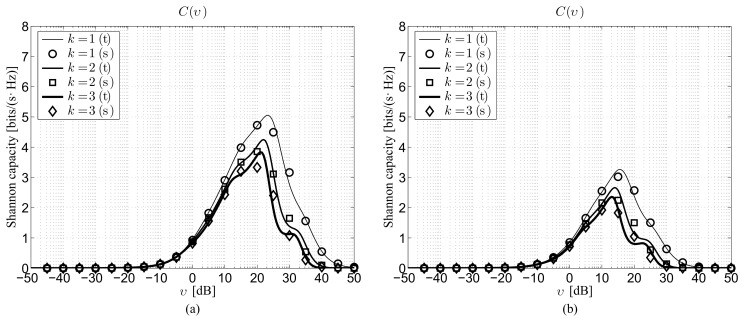
Theoretical and simulated Shannon capacity under perfect beam alignment for ρ=100 m and G=10 dB as a function of the SNR: (**a**) 28 GHz channel, (**b**) 73 GHz channel (t: theory, s: Monte Carlo simulation).

Figure 1 reports the Shannon capacity under perfect beam alignment for the first three *k* values considering ρ=100 m and G=10 dB in the 28 GHz (Figure 1a) and 73 GHz (Figure 1b) bands. Note that, under perfect beam alignment, i.e., Δ=1, (27) and (28) lead simply to:

(33) $$f_{G}\left(g\right)=\delta \left(g-G^{2}\right),$$

thus the backlobe gain *g* is not involved in the calculations. In the figures, the results derived from the proposed theoretical model are identified by lines, while markers are used to illustrate the corresponding performance obtained by independent Monte Carlo simulations. This second evaluation strategy, which relies on the execution of M= 100,000 realizations for each depicted point, is introduced to verify the accuracy of the analysis, with specific attention to the effects due to the usage of the improved Gaussian approximation and to the removal of the correlation between path loss attenuation and link state probability. The comparison between theoretical curves and simulations reveals a satisfactory reliability of the conceived model, thus confirming the acceptability of the two approximations. From the performance point of view, the figure shows that, for a given neighbor order, the lower 28 GHz band is preferable in terms of *C* because of the lower path loss attenuation with respect to the 73 GHz one (Table 1). As expected, for a given mmWave frequency, the higher the *k* value, the lower the capacity, since the $S-D_{k}$ distance statistically increases with the increase of the neighbor order. Naturally, the highest performance is achieved when $D_{k}=D_{1}$, i.e., when the destination is just the closest neighbor, while the difference between the capacities corresponding to two consecutive neighbor orders gets lower as *k*(≥2) gets higher.

### 4.2. Impact of Cell Radius

**Figure 2:**
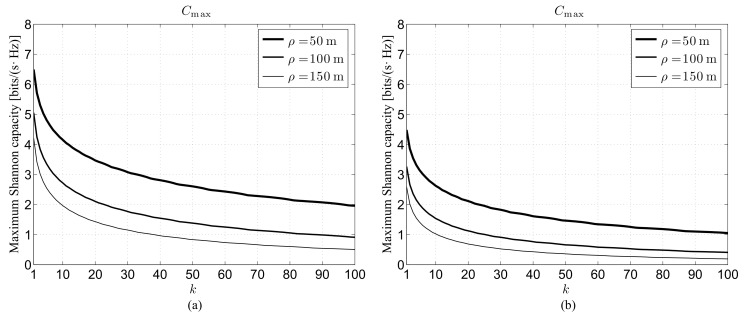
Maximum link capacity under perfect beam alignment for G=10 dB and different cell radii as a function of the neighbor order: (**a**) 28 GHz channel, (**b**) 73 GHz channel.

The second set of results is reported in Figure 2, which shows, under perfect beam alignment, the maximum Shannon capacity:

(34) $$C_{\max}=\max_{\upsilon \in \left(0,+\infty \right)}C\left(\upsilon \right),$$

as a function of the neighbor order for G=10 dB and different cell radii in the 28 GHz (Figure 2a) and 73 GHz (Figure 2b) frequency bands. From now on, the simulations will be no more inserted in order to simplify the readability of the figures. The displayed curves confirm that, given the mmWave channel and the neighbor order, a lower cell radius leads to a higher $C_{\max}$ value, suggesting the preferability of small cells for the 5G network deployment. This characteristic can be explained remembering that, according to (32), the increase of ρ leads to a reduction of the node intensity and, in turn, to a probabilistic increase of the source destination distance, similarly to what has been previously discussed concerning the impact of the *k* value. Besides, a direct comparison between Figure 2a,b reveals not only the expected higher performance for the lower mmWave band, but also the larger sensitivity of $C_{\max}$ with respect to *k* in the same band. Moreover, considering a given band and a given cell radius, the curves confirm, and also highlight, the smoothing of the performance decrease when the neighbor order increases.

### 4.3. Impact of Maximum Product Gain

**Figure 3:**
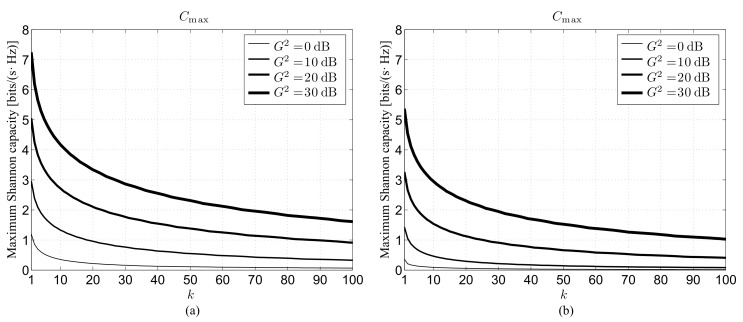
Maximum link capacity under perfect beam alignment for ρ=100 m and different maximum product gains $G^{2}$ as a function of the neighbor order: (**a**) 28 GHz channel, (**b**) 73 GHz channel.

The third set of results is illustrated in Figure 3, which presents the maximum Shannon capacity under perfect beam alignment as a function of the neighbor order for ρ=100 m and different maximum product gains $G^{2}$, still distinguishing between the 28 GHz (Figure 3a) and 73 GHz (Figure 3b) bands. The first aspect that may be observed from this novel figure concerns the need of using directional antennas to obtain an acceptable performance also in a 3D scenario. The adoption of omnidirectional antennas, corresponding to $G^{2}=0$ dB, leads in fact to a quick decrease of the maximum capacity towards zero even for moderate values of *k*. The case corresponding to the upper $G^{2}=30$ dB value might be instead viewed as a limiting situation when one takes into account the space available on the 5G devices for their transmitting/receiving antenna systems. Arrays consisting of a number of elements in the order of ten, capable of generating a maximum gain close to 10 dB, may be in fact assumed suitable for mobile equipment (ME), in which the area available for the antenna system is limited. Differently, arrays with one hundred elements, able to produce a maximum gain near 20 dB, might be considered possible for a BS, in which the available area is larger. Moreover, this ME–BS antenna configuration is able to maintain an acceptable capacity, including for very large neighbor orders in both mmWave bands. This capability becomes specifically relevant when one considers that, in agreement with the adopted three-state link model in (3), the results provided by Figure 3 are obtained, not only the LoS case, but also the more problematic OUT and NLoS ones.

### 4.4. Impact of Beam Alignment Error

**Figure 4:**
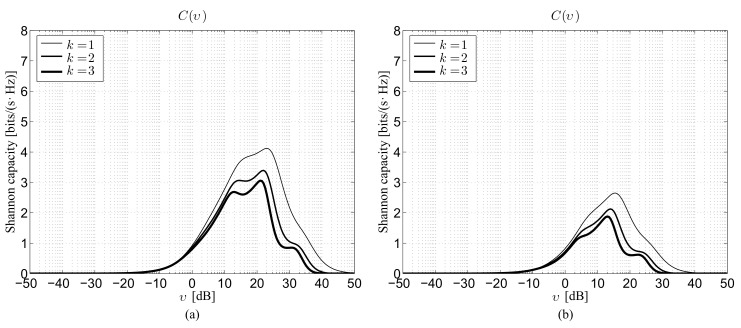
Theoretical Shannon capacity obtained in the presence of beam alignment error for ρ=100 m, G=10 dB, and g=0 dB as a function of the SNR: (**a**) 28 GHz channel, (**b**) 73 GHz channel.

The fourth set of results is shown in Figure 4, which depicts the Shannon capacity as a function of the SNR in the presence of a beam alignment error for ρ=100 m, G=10 dB, and g=0 dB, still considering the 28 GHz (Figure 4a) and 73 GHz (Figure 4b) bands. The curves are derived by choosing $\Omega =3\hat{\sigma }$ in (8) [6], thus obtaining $\Delta =\mathrm{erf}[3/\left(2\sqrt{2}\right)]≅0.87$. The influence of the pointing error on the link performance can be more clearly inferred by comparing this novel figure with the initial one reported in Section 4.1 (Figure 1). This comparison reveals that, for both bands and for all neighbor orders, the beam alignment error can approximately determine a 20% reduction of the Shannon capacity, with respect to the ideal case of perfectly steered antenna beams. In practical mmWave scenarios, such a performance decrease is expected to actually occur, because of the difficulty in aligning the radiation patterns of the transmitter and of the receiver when the patterns themselves are characterized by very narrow main lobes. Basically, the availability of many antenna elements at the BS or at the ME may provide a significant versatility in the generated pattern, since several degrees of freedom, i.e., synthesizable excitations, can be exploited. Moreover, a large number of array processing algorithms currently exist for providing any kind of shape; thus, the generation of wide or narrow main beams does not represent a problem. However, the current 5G cellular context, similar to the previous 2–4G ones, has to keep, as a priority, the control of the interference in directions that are not of interest, both for energy saving purposes and communication quality maintenance. Therefore, the usage of narrow beams represents an indispensable constraint. The adoption of wider beams would even potentially reduce the alignment problem, but at the cost not only of an interference increase, but also of a maximum gain reduction and, in turn, of a system coverage limitation.

### 4.5. Impact of Modulation

**Figure 5:**
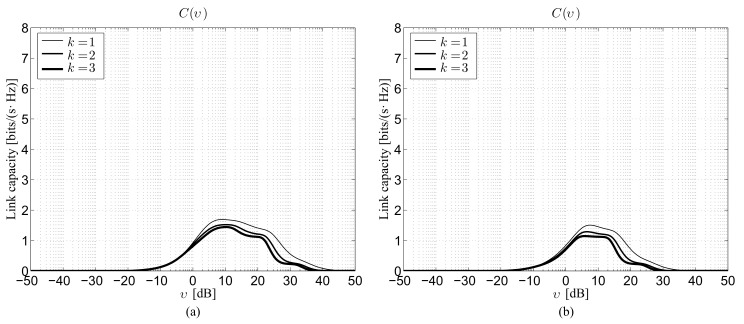
Theoretical capacity obtained using the QPSK modulation in the absence of beam alignment error for ρ=100 m and G=10 dB as a function of the SNR: (**a**) 28 GHz channel, (**b**) 73 GHz channel.

The fifth set of results is reported in Figure 5, which depicts, for the 28 GHz (Figure 5a) and 73 GHz (Figure 5b) bands, the link capacity as a function of the SNR under perfect beam alignment by considering ρ=100 m and G=10 dB. The significance of this latter figure can still be better understood by a direct comparison with Figure 1. The most evident element that can be noticed from this comparison is represented by the considerable gap between the Shannon capacity, achievable just adopting an ideal; that is, perfectly adaptive, modulation, and the link capacity actually obtainable through a fixed QPSK scheme. The performance decrease is in fact larger than 50%, even if the communications in the 28 GHz band seem to experience a more significant downgrade with respect to those carried out in the 73 GHz one. Accordingly, in this latter scenario, the $C_{\max}$ values for the two bands corresponding to a given neighbor order *k* become very close.

## 5. Conclusions

A 3D Poisson-based mathematical analysis for evaluating the link capacity of a source destination mmWave link has been proposed, with a specific focus on the impact of the neighbor order in the presence of path loss attenuation and mid-scale fading. The developed framework, which provides closed-form expressions, was validated by independent simulations and exploited to study the influence of the cell radius, of the maximum product antenna gain and of the frequency band on the maximum achievable performance, considering a general three-state link model.

The numerical results have revealed that, as long as the neighbor order is low, the capacity remains significant, while it quickly decreases for larger orders, finally approaching a limited low-varying performance for very high orders. This behavior was observed for the 28 and 73 GHz channels, in which the capacity improved when the cell radius was reduced and/or the maximum product gain was increased. A moderate decrease of the performance was noticed in the presence of beam alignment errors, while a much more significant one was checked when a realistic QPSK modulation was considered in place of the ideal Shannon bound. As a final comment, we should put into evidence the low computational burden required to implement the derived theoretical formulas, which, thanks to the routines available for the estimation of the special functions, have allowed a quick calculation of the analytical curves.

## Figures and Tables

**Table 1:** Adopted parameters [2].

$a_{\mathrm{out}}$	33.3 mm^−1^	$\alpha_{1}$	61.4 dB (28 GHz)	$\beta_{2}$	2.92 (28 GHz)
$b_{\mathrm{out}}$	5.2		69.8 dB (73 GHz)		2.69 (73 GHz)
$a_{\mathrm{los}}$	14.9 mm^−1^	$\alpha_{2}$	72.0 (28 GHz)	$\sigma_{1}$	5.8 dB (28 GHz)
$P_{S}$	0.1 W		82.7 (73 GHz)		5.8 dB (73 GHz)
*W*	1 GHz	$\beta_{1}$	2 (28 GHz)	$\sigma_{2}$	8.7 dB (28 GHz)
F	10 dB		2 (73 GHz)		7.7 dB (73 GHz)

## Abbreviations

The following abbreviations are used in this manuscript:

BS	base station
CCDF	complementary cumulative distribution function
CDF	cumulative distribution function
LoS	line of sight
mmWave	millimeter wave
ME	Mobile Equipment
NLoS	Non line of sight
OUT	OUTage
PDF	probability density function
PMF	probability mass function
PPP	Poisson point process
RV	random variable
SNR	signal to noise ratio
w.p.	with probability
